# Impact of Visit-to-Visit Triglyceride-Glucose Index Variability on the Risk of Cardiovascular Disease in the Elderly

**DOI:** 10.1155/2022/5125884

**Published:** 2022-09-16

**Authors:** Fei Chen, Ying Pan, Ziqing Liu, Rong Huang, Jing Wang, Jian Shao, Yaqin Gong, Xiyi Sun, Xiaobo Jiang, Weihao Wang, Zhaoqiang Li, Shao Zhong, Qi Pan, Kaixin Zhou

**Affiliations:** ^1^College of Life Sciences, University of Chinese Academy of Sciences, China; ^2^Department of Endocrinology, Kunshan Hospital Affiliated to Jiangsu University, Kunshan, Jiangsu, China; ^3^Guangzhou Laboratory, Guangzhou, Guangdong, China; ^4^Department of Medical Informatics, Kunshan Hospital Affiliated to Jiangsu University, Kunshan, Jiangsu, China; ^5^ZhenChuan Community Health Service Center, Kunshan, Jiangsu, China; ^6^Department of Endocrinology, Beijing Hospital, National Center of Gerontology, Institute of Geriatric Medicine, Chinese Academy of Medical Sciences, Beijing, China

## Abstract

**Background:**

The aging population is increasingly susceptible to cardiovascular disease (CVD). Visit-to-visit variability in glucose and lipid levels both contributed to CVD risk independent of their mean values. However, whether variability in the triglyceride-glucose (TyG) index is a risk factor for CVD remains unknown. *Research Design and Methods*. In this retrospective study of electronic health records, 27,520 participants aged over 60 years were enrolled. The visit-to-visit variability of TyG index was calculated from annual health examination data and defined as average real variability (ARV), standard deviation (SD), or the coefficient of variability (CV). CVD events were identified from the chronic disease registry or follow-up database and included myocardial infarction, angina, coronary, and stroke. Multivariate Cox regression was used to examine the correlation between TyG variability and incident CVD.

**Results:**

Over a median follow-up of 6.2 years, there were 2,178 CVD events. When participants were divided into four quartiles according to their TyG variability, after adjusting for established CVD risk factors, subjects in the top quartile had (HR = 1.18, 95% CI 1.05–1.34, *P*=0.005) significantly higher CVD risk than those in the bottom quartile. The association remained significant in overweight individuals or those without diabetes (*P* < 0.005 and *P* < 0.01, respectively).

**Conclusions:**

High variability in TyG was significantly associated with elevated CVD risk in the elderly, independent of average TyG and other risk factors. Close monitoring variability in TyG might be informative to identify old individuals at high risk of CVD.

## 1. Introduction

Cardiovascular diseases (CVD), which mainly include coronary artery disease (CAD) and stroke, are one of the leading causes of morbidity and mortality worldwide, accounting for 40% of death in China [1]. Established risk factors include family history, diabetes, obesity, hypertension, hypercholesteremia, and age, making CVD a major public health challenge for the aging population [2].

Measurable global progress has been made in the prevention of CVD within the last decade largely due to healthy lifestyle (smoking, physical activity, diet, and weight) [3]. Although a healthy lifestyle has been proven as an effective approach to CVD prevention, it remains inadequate due to the incomplete understanding of CVD pathophysiology and inaccurate population risk profiling [4].

Insulin resistance (IR), which is linked to most CVD risk factors, plays a key pathogenic role in the development of CVD [5]. However, its utility as a biomarker for CVD risk prediction has been sparse in the primary care setting [5], mainly due to the fact that accurate measurements of IR, such as the homeostasis model assessment of insulin resistance (HOMA-IR) and the total glucose metabolism rates from the hyperinsulinaemic–euglycaemic clamp, are complex and expensive to acquire. On the other hand, the triglyceride-glucose (TyG) index, which is the product of fasting plasma glucose (FPG) and triglyceride (TG), has been widely available and shown to be well correlated with the established parameters of IR [6, 7]. Consequently, a flurry of investigations convincingly demonstrated that the TyG index was associated with the risk of CVD, adding more predictive information to the traditional risk models [8–12]. However, none of the previous studies focused on the elderly in whom IR is more characterized by increasing physical inactivity and elevated peripheral resistance to insulin action as compared to those in the general population [13].

Recently, the visit-to-visit variability of CVD risk factors, evaluated as average real variability (ARV), standard deviation (SD), or the coefficient of variability (CV), gained more focus as a novel type of biomarker. The long-term individual variability in BMI, glucose, blood pressure, and lipid levels had been associated with CVD outcomes independent of their mean values [14–18]. Although the mechanisms by which such variability affected the risk of CVD remain largely elusive, most studies pointed to a potential role of IR as a common cause due to the fact that these risk factors are all correlated with IR to some extent [19]. However, whether the variability in TyG, the most readily available IR proxy, is an independent risk factor of CVD remains unknown.

We hypothesized that both the average value and the variability of TyG index are risk factors for CVD in the elderly. In this study, we assembled a large cohort of Chinese individuals aged over 60 and examined whether the mean and variability of TyG index were independently associated with incident CVD.

## 2. Research Design and Methods

### 2.1. Study Design and Participants

This is a population-based retrospective cohort study of electronic health record (EHR) linked data. A total number of 49,898 subjects were recruited from community health centers in Kunshan county, Jiangsu Province, China, between May 2019 and August 2021, while they took the annual health examinations which were offered free to the local elderly population. All participants signed informed consent to contribute their electronic health records dating back to 2014 for investigations aiming to improve the health of the elderly. Thus, we could rely on the visit-to-visit variability of TyG index through annual health examinations to examine its association with CVD incidence reported in the EHR. Details of the protocol for the current study were approved by the institutional review board of the First People's Hospital of Kunshan (IEC-C-007-A07-V3.0).

**Figure 1:**
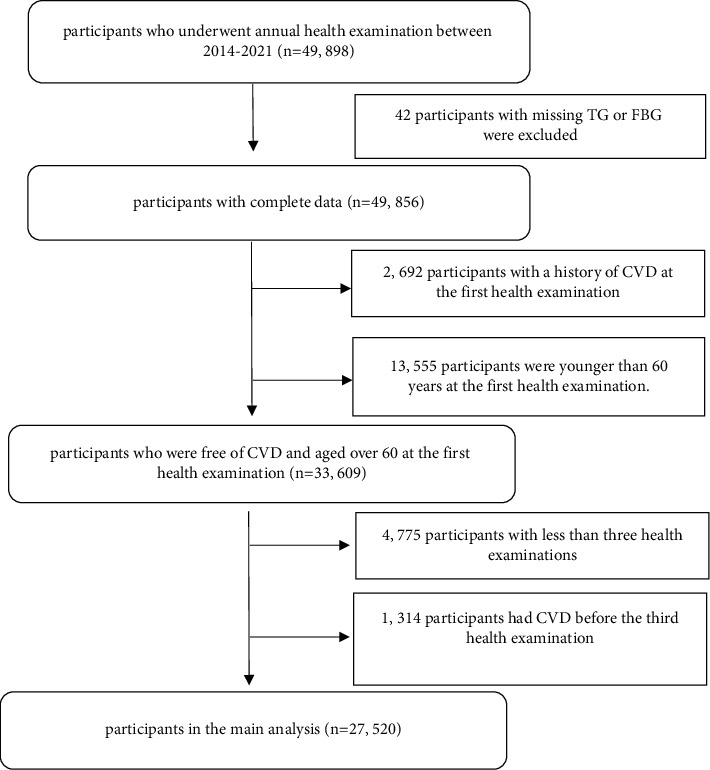
Flow chart of the study participants.

Of the 49,898 individuals who underwent annual health examinations between 2014 and 2021, we excluded those who had missing data of TG or FPG, a known history of CVD or were aged less than 60 at the time of the first health examination, less than three health examinations, and an incident CVD event prior to the third health examination. Finally, 27,520 participants were included in this study (Figure 1).

### 2.2. Data Source

For the current study, records from four databases were retrieved via the integrated regional health informatics system which covered all aspects of the public health and medical care services managed by the local health commission. The health examination database recorded a comprehensive lifestyle and quality questionnaire as well as routine physical function tests taken annually for the elderly. Simultaneously, a set of blood and urine biochemistry tests including fasting plasma glucose, lipid levels, liver enzymes, urine, and cancer biomarkers were also conducted and recorded annually. The chronic disease registry database recorded the onset of five common noncommunicable diseases, including diabetes, hypertension, coronary artery disease, stroke, and cancer. The chronic disease follow-up database kept the management details and outcomes on those individuals within the chronic disease registry. The current study also utilized the prescribing and biochemistry databases from the outpatient clinics to supplement the above databases for more accurate definitions of CVD outcomes.

### 2.3. CVD Outcome

The outcome evaluated in this study was the first occurrence of CVD. The types of CVD mainly included CAD (ICD-10 codes I20 to I25) and stroke (ICD-10 codes I60 to I64) as reported in the chronic disease registry or follow-up database. CAD was defined as hospital-admitted myocardial infarction or angina or coronary revascularization. Stroke was defined as ischemic or hemorrhagic stroke confirmed by computed tomography or magnetic resonance imaging. Incidence of CVD was also supplemented by the death registry in which the underlying cause of death was reported as CVD-related.

### 2.4. CVD Risk Factors and TyG Variability

For each individual, the first appearance in the health examination databases was defined as the index date. Baseline demographic characteristics such as sex, age, height, weight, exercise, and smoking status of the individuals were taken at this point. On the same day, a spectrum of other known CVD risk factors such as waist, systolic blood pressure (SBP), diastolic blood pressure (DBP), FPG, TG, high-density lipoprotein cholesterol (HDL-C), and low-density lipoprotein cholesterol (LDL-C) were measured and recorded at the community health centers. The TyG index was calculated as ln (fasting TG (mg/dL) × FPG (mg/dL)/2).

For the assessment of TyG variability, three consecutive measurements starting from the index date and prior to the first occurrence of CVD were used. The primary metric of variability was the ARV calculated as the average absolute difference between successive TyG measurements [20]. Two other commonly used metrics of variability, standard deviation (SD) and coefficient of variation (CV), were also calculated based on the same three TyG measurements.

### 2.5. Statistical Methods

The characteristics of individuals were presented as the mean (standard deviation) for quantitative parameters and percentage for categorical variables. The participants were stratified into four groups according to the quartiles of their TyG variability levels, and their characteristics were compared across these quartiles using ANOVA or *χ*^2^ test, as appropriate. The incidence rates of CVD were calculated by dividing the number of incident cases by the total follow-up period (person-years). The cumulative incidence of CVD events by quartiles of TyG variability was calculated, and the log-rank test was performed to confirm differences across the groups.

The association between TyG variability and CVD incidence was examined by multivariate Cox regression. Two models were assessed, with the basic model adjusted for age and sex only, and the full model is also adjusted for other established CVD risk factors. The likelihood ratio test for trends was used to elucidate a relationship between each quartile of TyG variability and the risk of CVD. The proportional hazard assumption in the Cox regression was assessed with the Schoenfeld residuals test [21].

We further conducted subgroups analyses in nondiabetic and overweight individuals. All statistical analyses were performed using R software version 4.1.0. The statistical significance level was set at a 2-tailed *P* < 0.05.

## 3. Results

### 3.1. Baseline Characteristics

Table 1 summarizes the baseline characteristics of the 27,520 participants according to the quartiles of their TyG variability as measured by ARV. The mean age of the participants was 66.8 years. The participants in the higher quartiles of TyG variability were younger, having elevated cardiometabolic risk profiles such as increased BMI, SBP, and mean TyG. A higher prevalence of diabetes and hypertension was also observed with increasing TyG variability. Similar associations existed with two other TyG variability measurements (SD and CV).

### 3.2. Higher TyG Variability Is Associated with More CVD Incidence

**Figure 2:**
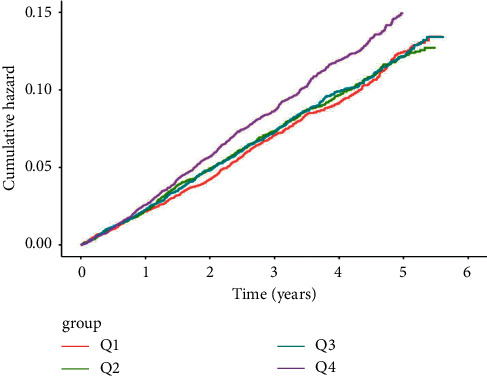
Cumulative incidence curves for the CVD events according to the quartiles of TyG variability.

Over a median follow-up of 6.2 years (interquartile range 4.2–7.1 years), there were 2,178 CVD events. When Cox proportional hazard models were used, elevated TyG variability, as measured by ARV, was significantly associated with higher CVD incidence with or without (*P* < 0.01 and *P* < 0.001) adjustment for known risk factors. Individuals in the top quartile had a significantly higher (HR = 1.31, 95% CI: 1.16–1.47) incidence of CVD compared to those in the bottom quartile in the basic model adjusted for age and sex only (Table 2). After adjusting for other established CVD risk factors, including the mean TyG, higher CVD incidence (HR = 1.18, 95%CI 1.05–1.34) was also observed in the top quartile compared to the bottom quartile (Table 2). Similar associations were observed when SD and CV were used to measure FPG variability. However, ARV is a more reliable representation of time series variability [22]; therefore, the downstream sensitivity analyses only showed the results of ARV. Cumulative incidence curves for the CVD events according to the quartiles of TyG variability are shown in Figure 2, and the *P* value of the log-rank test for the trend was 0.00035.

### 3.3. Sensitivity Analyses

#### 3.3.1. TyG Variability Is Associated with CVD in Nondiabetic Individuals

In the sensitivity analysis restricted to participants without diabetes (*n* = 24,121), higher TyG variability was significantly associated (*P* < 0.01) with more CVD events after adjusting for known risk factors. Compared to the bottom quartile, higher CVD incidence (HR = 1.2, 95% CI 1.05–1.37) was observed in the top quartile, demonstrating TyG variability was an independent CVD risk factor for individuals without diabetes.

#### 3.3.2. TyG Variability Is Associated with CVD in Overweight Individuals

In overweight (BMI >24 kg/m^2^, *n* = 14,660) individuals, increased TyG variability was significantly (*P* < 0.05) associated with more CVD events after adjusting for known risk factors. The HR for CVD events was 1.21 (95% CI 1.03–1.41) for the top quartile compared with the bottom quartile, suggesting TyG variability was an independent CVD risk factor for the overweight individuals.

## 4. Conclusions

In this large study of community based elderly participants, after adjusting for the average level of TyG index, we observed individuals with elevated visit-to-visit TyG variability, especially those in the top quartile had significantly more CVD events. Such an association remained in overweight participants or among those without diabetes, suggesting long-term TyG variability could be an important indicator of elevated CVD risk for the elderly.

There are multiple ways to characterize the variability in the TyG index. A recent study using pattern recognition showed that normal weight individuals were typically on one of the five different trajectories of TyG index over a period of time, and each trajectory marked variable risk of CVD, with those on the ascending TyG trend bearing highest risk of future CVD [23]. The pattens of trajectories were challenging to interpret as the contribution to CVD risk from mean and variability of TyG which could not be differentiated. Here, we used common matrices such as SD, CV, and ARV to quantify the visit-to-visit variability in TyG. We were able to show that all these quantitative measures of variability in TyG were significantly associated with CVD risk independent of the average TyG. The main variability parameter ARV reported in this study is particularly robust when only a small number of TyG measurements are available, and it is orthogonal to the trend within the time series data [22]. Thus, our results provided a solid foundation for replication and in-depth investigation into the role of TyG variability in CVD.

Although we observed an overall association between TyG variability and CVD incidence, it is worth noting the association was mainly driven by those within the top quartile of TyG variability. Within this quartile, there was a significant enrichment of patients with diabetes as shown in Table 1. Therefore, we performed a sensitivity analysis in the subgroup without diabetes. In spite of lower TyG variability in these participants without diabetes, individuals within the top quartile of TyG variability remained significantly associated with more CVD incidence. Therefore, the association between TyG variability and CVD was pervasive in the elderly regardless of preexisting diabetes.

Another sensitivity analysis was performed within the overweight individuals. This is mainly due to the fact that the previous study demonstrating a link between TyG trajectory and CVD risk was confined to the normal weight population [23]. Here, we found the participants in the top quartile of TyG variability also had higher BMI. Our result extended upon the previous study by confirming within the overweight individuals that higher TyG variability remained an independent CVD risk factor.

Our investigation utilized a cohort of elderly participants with higher CVD burden. Among them, elevated TyG variability was associated with a broad high CVD risk profile including elevated BMI, blood pressure, glucose, and lipids levels, all of which might be indicative of IR. Such results are consistent with previous studies demonstrating higher TyG variability was associated with increased CVD risk in the elderly [24]. With the natural process of aging, the elderly was prone to IR due to reduction in physical activity. Moreover, aging related progressive deterioration in structure and function of the heart and vasculature may also contribute to the development of CVD [25]. Therefore, whether TyG variability is associated with CVD in the younger adult population remains to be elucidated. Given that TyG is a routinely available surrogate of IR, our results suggest that potential health benefit could be gained by closely monitoring TyG variability in the elderly.

Our findings are important because they could provide important implications for the prevention of and intervention for CVD. Our results provide evidence that the long-term TyG variability can be an important indicator for predicting CVD among the elderly. This finding suggests that monitoring variability of TyG index may provide an important approach to identify individuals with higher risk of CVD and help to prevent primary CVD in the elderly. Furthermore, the finding remained in higher risk population with diabetes or overweight participants. This finding indicates a rapid increase in TyG variability in higher risk population before the onset of disease contributed more to the pathological process of CVD, which emphasize the importance of maintaining a stable level of TyG variability in the prevention of CVD. From another perspective, our results indicate that a large proportion of participants with new-onset CVD experienced a rapid increase in TyG variability before the onset of disease, which emphasize the important role of TyG variability in the development of CVD.

The biological mechanisms underlying the association between TyG variability and CVD is largely unknown. At the molecular level, IR has been well established to induce endothelial dysfunction [26], oxidative stress [27], cardiovascular remodeling [28], and inflammation response [19]. Whilst FPG and TG mainly reflect IR in the liver and adipose tissue, respectively [29, 30]. TyG as their composite is a better proxy of multisystem IR. That is why, TyG has been consistently linked to a spectrum of CVD risk factors such as arterial stiffness and coronary artery calcification [31–33]. With regard to the risk specific to variability, it is possible that fluctuations in IR may lead to more heterogeneous composition of vascular plaques vulnerable to rapture [34], endothelial function [35], and inflammatory response [36].

Here, we conducted an observational study of the association between TyG variability and CVD incidence. The assembly of such a large community-based cohort for sufficient statistical power took the advantage of comprehensive EHR data from the integrated local health care record system. The freely available annual health examination to all the elderly also made our cohort more robust to sample ascertainment bias and ensured consistent TyG variability assessment. However, due to the nature of real-world health care data, no research-oriented IR data such as the HOMA-IR were available to better examine its link with CVD. Although we adjusted the effects of diabetes and hypertension on CVD, we did not consider other diseases of the elderly, such as arthritis and liver failure. The main reason was that no relevant records in EHR. Moreover, our study could not differentiate the TyG variability owing to natural fluctuation from that driven by drug treatment mainly due to the highly complex level of multiple medications existing in this elderly cohort.

We found that a high TyG variability was more closely associated with CVD incident independent of the average TyG index, which suggests that TyG variability can be an important indicator for predicting CVD among the elderly. Furthermore, the sensitivity analysis in overweight participants or among those without diabetes remained the association, suggesting the results are robust. The analysis in the overweight participants extend the previous study demonstrating that the association between TyG variability and CVD incident applies to normal weight and overweight. The association between the TyG variability and CVD incident among those without diabetes indicating that individuals need to pay attention to the TyG variability even without diabetes.

In conclusion, we found that, in the elderly population, high TyG variability was associated with elevated risks of CVD independent of the average TyG index. Further studies are warranted to establish whether close monitoring or intervention to reduce TyG fluctuation could result in better health outcome.

## Figures and Tables

**Table 1:** Baseline characteristics according to the quartiles of TyG variability.

	Total	*Q*1 (0–0.17)	*Q*2 (0.17–0.27)	*Q*3 (0.27–0.42)	*Q*4 (0.42–3.03)	*P*
Demographic characteristics						
*N*	27,520	6,880	6,880	6,880	6,880	
Age, years	66.8 (5.2)	67.2 (5.3)	66.9 (5.2)	66.7 (5.2)	66.2 (4.9)	<0.001
Female	14,232 (52%)	3,450 (50%)	3,520 (51%)	3,576 (52%)	3,686 (54%)	0.14
Waist, cm	83.5 (8.8)	83.3 (9.0)	83.4 (8.7)	83.5 (8.9)	83.8 (8.6)	0.006
BMI, kg/m^2^	24.4 (3.4)	24.3 (3.4)	24.3 (3.3)	24.4 (3.5)	24.6 (3.4)	<0.001
Smoking statue						0.14
Ever smoking	6,209 (23%)	1,586 (23%)	1,599 (23%)	1,518 (22%)	1,506 (22%)	
Never	21,311 (77%)	5,294 (77%)	5,281 (77%)	5,362 (78%)	5,374 (78%)	
Exercise status						0.04
Not active	15,706 (57%)	3,842 (56%)	3,928 (57%)	3,961 (58%)	3,975 (58%)	
Somewhat active	2,724 (10%)	672 (10%)	665 (10%)	667 (10%)	720 (10%)	
Regularly active	9,090 (33%)	2,366 (34%)	2,287 (33%)	2,252 (33%)	2,185 (32%)	
Clinical characteristics						
Diabetes	3,399 (12%)	616 (9%)	687 (10%)	871 (13%)	1,225 (18%)	<0.001
Hypertension	12,893 (47%)	3,044 (44%)	3,160 (46%)	3,226 (47%)	3,463 (50%)	<0.001
SBP, mmHg	139.6 (19.0)	139.1 (19.1)	139.3 (18.9)	139.7 (18.8)	140.3 (19.0)	0.002
DBP, mmHg	80.6 (10.5)	80.3 (10.7)	80.4 (10.6)	80.6 (10.3)	80.9 (10.3)	0.005
LDL-C, mmol/L	2.8 (0.9)	2.8 (0.9)	2.8 (0.9)	2.9 (0.9)	2.8 (0.9)	0.024
HDL-C, mmol/L	1.47 (0.5)	1.51 (0.6)	1.48 (0.5)	1.48 (0.5)	1.43 (0.5)	<0.001
Mean TyG (mg/dL)^2^	8.74 (0.5)	8.64 (0.5)	8.68 (0.5)	8.74 (0.5)	8.92 (0.6)	<0.001

Data are presented as mean (SD) for continuous variables or *n* (%) for categorical variables. *Q*, quartile; BMI, body mass index; SBP, systolic blood pressure; DBP, diastolic blood pressure; LDL-C, low-density lipoprotein cholesterol; HDL-C, high-density lipoprotein cholesterol; TyG, triglyceride-glucose.

**Table 2:** The associations between TyG variability quartiles and CVD.

Quartiles	Event (*n*)	Follow-up duration (Person-years)	Incidence rate (per 1000 person-years)	Basic model		Full model	
				HR (95% CI)	*P* value	HR (95% CI)	*P* value
*Q*1	508	37,871	13.41	Reference		Reference	
*Q*2	509	37,703	13.5	1.03 (0.91,1.17)	0.62	1.01 (0.89,1.14)	0.90
*Q*3	520	38,150	13.63	1.04 (0.92,1.18)	0.49	1 (0.88,1.13)	0.99
*Q*4	641	38,678	16.57	1.31 (1.16,1.47)	<0.001	1.18 (1.05,1.34)	0.005
*P* for trend				<0.001		<0.01	

Basic model: adjusted for sex and age, full model: adjusted for sex, age, BMI, SBP, HDL-C, LDL-C, hypertension, diabetes, and mean TyG.

## Data Availability

The datasets used in the study are available from the corresponding author upon reasonable request.

## References

[1] Zhou M., Wang H., Zeng X. (2019). Mortality, morbidity, and risk factors in China and its provinces, 1990–2017: a systematic analysis for the global burden of disease study 2017. *Lancet*.

[2] Mendis S. (2017). Global progress in prevention of cardiovascular disease. *Cardiovascular Diagnosis and Therapy*.

[3] Virani S. S., Alonso A., Benjamin E. J. (2020). Heart disease and stroke statistics—2020 update: a report from the American heart association. *Circulation*.

[4] Cowie A., Buckley J., Doherty P. (2019). Standards and core components for cardiovascular disease prevention and rehabilitation. *Heart*.

[5] Ormazabal V., Nair S., Elfeky O., Aguayo C., Salomon C., Zuñiga F. A. (2018). Association between insulin resistance and the development of cardiovascular disease. *Cardiovascular Diabetology*.

[6] Sánchez-García A., Rodríguez-Gutiérrez R., Mancillas-Adame L. (2020). Diagnostic accuracy of the triglyceride and glucose index for insulin resistance: a systematic review. *International Journal of Endocrinology*.

[7] Mazidi M., Kengne A.-P., Katsiki N., Mikhailidis D. P., Banach M. (2018). Lipid accumulation product and triglycerides/glucose index are useful predictors of insulin resistance. *Journal of Diabetes and Its Complications*.

[8] Ding X., Wang X., Wu J., Zhang M., Cui M. (2021). Triglyceride–glucose index and the incidence of atherosclerotic cardiovascular diseases: a meta-analysis of cohort studies. *Cardiovascular Diabetology*.

[9] Barzegar N., Tohidi M., Hasheminia M., Azizi F., Hadaegh F. (2020). The impact of triglyceride-glucose index on incident cardiovascular events during 16 years of follow-up: tehran lipid and glucose study. *Cardiovascular Diabetology*.

[10] Luo E., Wang D., Yan G. (2019). High triglyceride–glucose index is associated with poor prognosis in patients with acute ST-elevation myocardial infarction after percutaneous coronary intervention. *Cardiovascular Diabetology*.

[11] Sánchez-Íñigo L., Navarro-González D., Fernández-Montero A., Pastrana-Delgado J., Martínez J. A. (2016). The TyG index may predict the development of cardiovascular events. *European Journal of Clinical Investigation*.

[12] Liu X. c., He G. d., Lo K., Huang Y. q., Feng Y. q. (2020). The triglyceride-glucose index, an insulin resistance marker, was non-linear associated with all-cause and cardiovascular mortality in the general population. *Front Cardiovasc Med*.

[13] Scheen A. J. (2005). Diabetes mellitus in the elderly: insulin resistance and/or impaired insulin secretion?. *Diabetes and Metabolism*.

[14] Nam G. E., Kim W., Han K. (2020). Body weight variability and the risk of cardiovascular outcomes and mortality in patients with type 2 diabetes: a nationwide cohort study. *Diabetes Care*.

[15] Li S, Nemeth I, Donnelly L, Hapca S, Zhou K, Pearson ER (2020). Visit-to-visit HbA _1c_ variability is associated with cardiovascular disease and microvascular complications in patients with newly diagnosed type 2 diabetes. *Diabetes Care*.

[16] Wan E. Y. F., Fung C. S. C., Yu E. Y. T., Fong D. Y. T., Chen J. Y., Lam C. L. K. (2017). Association of visit-to-visit variability of systolic blood pressure with cardiovascular disease and mortality in primary care Chinese patients with type 2 diabetes—a retrospective population-based cohort study. *Diabetes Care*.

[17] Lee E. Y., Yang Y., Kim H.-S. (2018). Effect of visit-to-visit LDL-HDL-and non-HDL-cholesterol variability on mortality and cardiovascular outcomes after percutaneous coronary intervention. *Atherosclerosis*.

[18] Lee S.-H., Kim M. K., Rhee E.-J. (2020). Effects of cardiovascular risk factor variability on health outcomes. *Endocrinology and Metabolism*.

[19] Laakso M., Kuusisto J. (2014). Insulin resistance and hyperglycaemia in cardiovascular disease development. *Nature Reviews Endocrinology*.

[20] Nwabuo C. C., Yano Y., Moreira H. T. (2020). Association between visit-to-visit blood pressure variability in early adulthood and myocardial structure and function in later Life. *JAMA Cardiol*.

[21] Lin D. Y., Wei L. J., Ying Z. (1993). Checking the cox model with cumulative sums of martingale-based residuals. *Biometrika*.

[22] Mena L., Pintos S., Queipo N. V., Aizpúrua J. A., Maestre G., Sulbarán T. (2005). A reliable index for the prognostic significance of blood pressure variability. *Journal of Hypertension*.

[23] Tian X., Zuo Y., Chen S. (2022). Distinct triglyceride-glucose trajectories are associated with different risks of incident cardiovascular disease in normal-weight adults. *American Heart Journal*.

[24] Zhao S., Yu S., Chi C. (2019). Association between macro- and microvascular damage and the triglyceride glucose index in community-dwelling elderly individuals: the Northern Shanghai Study. *Cardiovascular Diabetology*.

[25] Donato A. J., Machin D. R., Lesniewski L. A. (2018). Mechanisms of dysfunction in the aging vasculature and role in age-related disease. *Circulation Research*.

[26] Muniyappa R., Chen H., Montagnani M., Sherman A., Quon M. J. (2020). Endothelial dysfunction due to selective insulin resistance in vascular endothelium: insights from mechanistic modeling. *American Journal of Physiology—Endocrinology And Metabolism*.

[27] Hurrle S., Hsu W. H. (2017). The etiology of oxidative stress in insulin resistance. *Biomedical Journal*.

[28] Yang C. D., Shen Y., Lu L. (2019). Insulin resistance and dysglycemia are associated with left ventricular remodeling after myocardial infarction in non-diabetic patients. *Cardiovascular Diabetology*.

[29] DeFronzo R. A., Ferrannini E., Groop L. (2015). Type 2 diabetes mellitus. *Nature Reviews Disease Primers*.

[30] Guilherme A., Virbasius J. V., Puri V., Czech M. P. (2008). Adipocyte dysfunctions linking obesity to insulin resistance and type 2 diabetes. *Nature Reviews Molecular Cell Biology*.

[31] Su Y., Wang S., Sun J. (2021). Triglyceride glucose index associated with arterial stiffness in Chinese community-dwelling elderly. *Front Cardiovasc Med*.

[32] Wu Z., Zhou D., Liu Y. (2021). Association of TyG index and TG/HDL-C ratio with arterial stiffness progression in a non-normotensive population. *Cardiovascular Diabetology*.

[33] Park K., Ahn C. W., Lee S. B. (2019). Elevated TyG index predicts progression of coronary artery calcification. *Diabetes Care*.

[34] Wu S., Liu W., Ma Q. (2019). Association between insulin resistance and coronary plaque vulnerability in patients with acute coronary syndromes: insights from optical coherence tomography. *Angiology*.

[35] Li M., Qian M., Xu J. (2017). Vascular endothelial regulation of obesity-associated insulin resistance. *Frontiers in Cardiovascular Medicine*.

[36] Zatterale F., Longo M., Naderi J. (2019). Chronic adipose tissue inflammation linking obesity to insulin resistance and type 2 diabetes. *Frontiers in Physiology*.

